# Brain Responses during the Anticipation of Dyspnea

**DOI:** 10.1155/2016/6434987

**Published:** 2016-08-28

**Authors:** M. Cornelia Stoeckel, Roland W. Esser, Matthias Gamer, Christian Büchel, Andreas von Leupoldt

**Affiliations:** ^1^Department of Systems Neuroscience, University Medical Center Hamburg-Eppendorf, Martinistraße 52, 20246 Hamburg, Germany; ^2^Department of Psychology 1, University of Würzburg, Marcusstraße 9-11, 97070 Würzburg, Germany; ^3^Research Group Health Psychology, University of Leuven, Tiensestraat 102, 3000 Leuven, Belgium

## Abstract

Dyspnea is common in many cardiorespiratory diseases. Already the anticipation of this aversive symptom elicits fear in many patients resulting in unfavorable health behaviors such as activity avoidance and sedentary lifestyle. This study investigated brain mechanisms underlying these anticipatory processes. We induced dyspnea using resistive-load breathing in healthy subjects during functional magnetic resonance imaging. Blocks of severe and mild dyspnea alternated, each preceded by anticipation periods. Severe dyspnea activated a network of sensorimotor, cerebellar, and limbic areas. The left insular, parietal opercular, and cerebellar cortices showed increased activation already during dyspnea anticipation. Left insular and parietal opercular cortex showed increased connectivity with right insular and anterior cingulate cortex when severe dyspnea was anticipated, while the cerebellum showed increased connectivity with the amygdala. Notably, insular activation during dyspnea perception was positively correlated with midbrain activation during anticipation. Moreover, anticipatory fear was positively correlated with anticipatory activation in right insular and anterior cingulate cortex. The results demonstrate that dyspnea anticipation activates brain areas involved in dyspnea perception. The involvement of emotion-related areas such as insula, anterior cingulate cortex, and amygdala during dyspnea anticipation most likely reflects anticipatory fear and might underlie the development of unfavorable health behaviors in patients suffering from dyspnea.

## 1. Introduction

Dyspnea is the aversive and threatening cardinal symptom in prevalent diseases such as asthma and chronic obstructive pulmonary disease (COPD) and associated with great individual and socioeconomic burden [1]. In chronic respiratory conditions the adequate perception of dyspnea plays a key role as it has a strong influence on health behavior and course of disease. Notably, the perception of dyspnea is not tightly related to objective lung function [2] but is modulated by cognitive and affective factors [3–6].

The few available neuroimaging studies investigating the neural processing of dyspnea [7–14] underline the importance of sensorimotor, cognitive, and emotion-related brain areas. A dual pathway model has been suggested [15, 16] with one pathway including ventroposterior thalamic areas and sensorimotor cortices processing the sensorimotor aspects of dyspnea. The second pathway including medial-dorsal thalamic areas, insula, amygdala, and cingulate cortex is believed to process the affective aspects of dyspnea. Of all these areas the paralimbic insula with its implication in interoceptive and emotion-related processing seems to play a key role [4, 11, 16, 17]. Notably, recent studies have demonstrated that negative emotions are related not only to increased perception but also to changes in the neural processing of dyspnea [18].

Patients suffering from chronic dyspnea tend to avoid discomfort by reducing daily-life physical activities. Activity avoidance results in progressive deconditioning, which further increases dyspnea [19]. In particular the fearful anticipation of dyspnea has been hypothesized to lead up to this spiral of decline [20]. Indeed, recent studies demonstrated that the anticipation of dyspnea is associated with increased physiological fear responses [21], especially in anxious individuals [22]. Although the fearful anticipation of dyspnea might play a fundamental role for disease progression the underlying brain processes have rarely been studied.

Investigations on the anticipation of pain, a similarly aversive bodily sensation, indicate that pain-sensitive areas are already activated during pain anticipation [23–25]. Moreover, brain activation during pain anticipation predicts and influences the subsequent perception [26] and neural processing of pain [27–29]. Anticipatory changes of brain function in areas with high importance for emotion processing such as insula, anterior cingulate cortex (ACC), amygdala, and midbrain/periaqueductal gray (PAG) were particularly relevant [26, 30, 31].

Similar mechanisms can be expected for the anticipation and perception of dyspnea. If brain activation during the anticipation of dyspnea would indeed influence and shape subsequent dyspnea perception, this might be particularly relevant for a better understanding of dyspnea avoidance behavior in patients suffering from chronic dyspnea and for the development of tailored treatment strategies.

Therefore, we used functional magnetic resonance imaging (fMRI) to investigate the brain processes underlying the anticipation of resistive-load-induced dyspnea in healthy volunteers. Specifically, we tested the hypotheses that the anticipation of dyspnea is processed in brain areas related to the perception of dyspnea and would involve prominent activations in emotion-related areas [18]. Moreover, we hypothesized that brain activation during dyspnea anticipation would relate to brain activation during subsequent dyspnea perception.

## 2. Materials and Methods

### 2.1. Participants

We recruited 46 healthy subjects without history of respiratory disease from a large database of genotyped individuals (Table 1). Genotype related differences concerning the neural processing of dyspnea as well as habituation effects have been reported elsewhere [32, 33] while the current analyses specifically focus on anticipatory processes. All data were collected on one day and normal lung function (forced expiratory volume in one second in % predicted > 80%) was confirmed by standard spirometry [34] on the day of the experiment. Written informed consent was obtained prior to the study. The study protocol was approved by the ethics committee of the Medical Association Hamburg (PV3662).

### 2.2. Apparatus and Respiratory Measurements

Volunteers breathed through a face mask connected with an MRI compatible pneumotachograph (ZAN 600 unit, ZAN Messgeräte GmbH, Oberhulba, Germany). The set-up contained ports for recording of end-tidal CO_2_ pressure (*P*_ET_CO_2___) and peak inspiratory mouth pressure (*P*_*I*_) and a two-way nonrebreathing valve. The inspiratory port of the valve was connected to a 2.6 m tube for the easy manual introduction and removal of MR-compatible resistive loads in the scanner environment by the experimenter. The free expiratory port prevented rebreathing of CO_2_. This breathing circuit allowed continuous measurements of respiratory parameters including *P*_ET_CO_2___, peak inspiratory pressure, tidal volume (*V*_*T*_), breathing frequency (*f*), minute ventilation (*V*_*E*_), and inspiratory time (*T*_*I*_).

### 2.3. Induction and Measurement of Perceived Dyspnea

We explained dyspnea to our participants as a sensation of difficult and uncomfortable breathing. In a pretest subjects were placed in a supine position and presented with inspiratory resistive loads of increasing magnitude. Each load was presented for 24 s and dyspnea intensity subsequently rated on a Borg-scale (0 = “not noticeable” to 10 = “maximally imaginable”). Load magnitude was increased until subjects reliably reported a sensation of “severe” dyspnea (Borg score ≥ 5). The respective load was then used to induce severe dyspnea during scanning (mean load resistance = 2.23 kPa/L/s, SD = 1.18). For the baseline condition of “mild” dyspnea the smallest resistive load that was reliably rated as different from unloaded breathing was used (mean load resistance = 0.25 kPa/L/s, SD = 0.18).

### 2.4. Instructions

Subjects learned the association of colored cues in the shape of a cross and experimental conditions both during a computer-based standardized instruction outside the scanner and during a short test run within the scanner where subjects were also acquainted with the button response system. Thus, subjects were well familiar with the cue, stimulus association prior to the acquisition of functional MRI data.

### 2.5. Experimental Protocol

**Figure 1:**
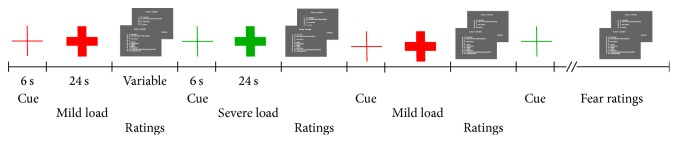
MRI task and protocol. In alternating order, subjects were visually cued for either mild or severe dyspnea followed by the respective load. Each load was followed by two Borg rating scales, one on dyspnea intensity and one on dyspnea unpleasantness. The order of the scales was randomized. Each anticipatory cue lasted for 6 s. Then the cue, a thin cross, turned into a solid cross and the load was introduced manually for 24 s. There were ten blocks of mild and severe dyspnea, respectively. The last intensity and unpleasantness ratings were followed by ratings on the average anticipatory fear during the two different cues.

Immediately after pretest and standardized instructions, subjects entered a 3-Tesla TRIO-Magnetom Scanner (Siemens, Medical Solutions, Erlangen, Germany) with the face mask tightly fitted. Visual cues and Borg-scales were projected into the scanner bore via a mirror and condition markers were sent to ZAN-system using Presentation software (Neurobehavioral Systems, Inc., Albany, CA). In alternating order, subjects were presented with the visual color-coded cue for either mild or severe dyspnea followed by the respective load (Figure 1). Each anticipatory period lasted for 6 s. Then the cue, a thin cross, turned into a solid cross and the preselected load was introduced manually for 24 s as in our previous fMRI studies using similar stimuli [11, 12]. There were 10 blocks of mild and 10 blocks of severe dyspnea. Each load was followed by two Borg rating scales: one on dyspnea intensity and one on dyspnea unpleasantness during the preceding block. The order of the scales was randomized. Following the final dyspnea ratings subjects were presented with two additional Borg-scales asking to indicate the level of fear experienced on average during the cue conditions (anticipation) of mild and severe dyspnea, respectively (Figure 1). Immediately following the brain scan subjects rated the perceived quality of dyspnea on a verbal descriptor list [35] outside the scanner.

### 2.6. fMRI Data Acquisition

Imaging was performed on a 3-Tesla TRIO-Magnetom Scanner (Siemens, Medical Solutions, Erlangen, Germany) using a standard 32-channel head-coil. For each data volume we acquired 48 continuous axial-slices in descending order with 2 × 2 mm in-plane resolution, 2 mm slice thickness, and a 1 mm gap using T2^*∗*^-weighted parallel echoplanar imaging (TR = 2870 ms, TE = 25 ms, flip angle = 80°, and field of view = 208 × 208 mm) with GRAPPA acceleration (*R* = 2). Depending on the time spent on ratings subjects needed 13–18 min to complete the protocol. The number of functional scans acquired varied accordingly (275–374 volumes). The first 5 volumes were discarded to allow for T1-saturation. Following fMRI, we acquired a high-resolution T1-weighted structural brain scan using a standard MP-RAGE sequence (1 × 1 × 1 mm spatial resolution, TR = 2300 ms, TE = 2.98 ms, flip angle = 9°, and field of view = 256 × 256, 240 slices).

### 2.7. Data Analysis

Means of intensity, unpleasantness, and anticipatory fear ratings were compared between mild and severe dyspnea conditions using paired *t*-tests. Respiratory parameters for each block and condition were analyzed as dependent variables in separate one-way repeated measures ANOVAs across the four conditions (anticipation mild, mild dyspnea, anticipation severe, severe dyspnea) followed by Bonferroni-corrected paired *t*-tests to further explore significant main effects. Analyses were calculated with SPSS 20.0 software (SPSS Inc., Chicago, IL) using a significance level of *p* < 0.05.

All steps of fMRI data preprocessing and statistical analysis were carried out using SPM8 (http://www.fil.ion.ucl.ac.uk/spm/), with the exception of noise-correction, which was carried out using fsl-MELODIC 3.0. From the ten presented blocks of each condition, the first two blocks of each condition (i.e., 2 × anticipation mild, 2 × mild dyspnea, 2 × anticipation severe, and 2 × severe dyspnea) served as adaptation phase and did not enter the analyses. A custom template within standard space was created from the T1 images of all participants using the DARTEL-protocol implemented within SPM8. Functional data were unwarped and realigned using 6-parameter rigid-body transformations. After normalization to the custom-made T1-template using linear and nonlinear transformations, noise was identified based on a probabilistic independent component analysis. Preprocessed data were whitened and projected into a 40-dimensional subspace using Principal Component Analysis and further decomposed into sets of vectors that describe signal variation across the temporal domain (time courses) and across the spatial domain (spatial maps) by optimizing for non-Gaussian spatial source distributions [36]. Each component was categorized as either function-related (resting-state networks or paradigm related) or noise-related (e.g., noise due to respiration, cardiac activity, motion, or scanner drifts) by considering the spatial pattern, the time course, and the power distribution following a heuristic described by Kelly et al. [37]. This procedure also controlled for potential effects of *P*_ET_CO_2___ fluctuations on brain activation. Two independent raters showed high interrater agreement (96.6%, Cohen's Kappa = 0.8). Components consistently identified as noise were filtered out. Noise-corrected functional data were smoothed using an 8 × 8 × 8 mm full-width at half-maximum Gaussian filter.

For statistical analysis data were high-pass filtered with a 128 s cut-off, while serial correlations were accounted for by using an autoregressive model. Data modeling on the first level involved separate regressors for each condition (cue mild, mild dyspnea, cue severe, severe dyspnea, and ratings) based on the canonical haemodynamic response function implemented in SPM8. Mean BOLD signal intensity of each image was included as regressor of no interest. On the subject-level we contrasted cue severe with cue mild and severe with mild resistive-load-induced dyspnea to extract brain areas that show more activation during the anticipation and perception of severe versus mild dyspnea, respectively. These two contrast images per subject were then entered into separate random-effects group analyses.

Next, we investigated how dyspnea-related brain areas that also showed activation during dyspnea anticipation interacted with other brain areas during the anticipation of severe as compared to mild dyspnea. For these psychophysiological interactions (PPI [38]), we extracted the average individual time courses from a volume centered on the peak voxel of the (anticipation severe versus anticipation mild) contrast for each of the investigated areas (left insula, parietal operculum, and cerebellum, see results) and used the anticipation of severe versus mild dyspnea as modulatory experimental factor.

Given the assumed prominent role of the insular cortex in processing the affective qualities of perceived dyspnea [11, 16, 39, 40], we investigated the influence of brain activation during dyspnea anticipation on individual average right insular activation during the subsequent perception of resistive-load-induced dyspnea. Individual parameter estimates from the perception contrast (severe dyspnea versus mild dyspnea) served as covariate for the anticipation contrast (anticipation severe dyspnea versus anticipation mild dyspnea).

Finally, we were interested how anticipatory fear is related to anticipatory brain activation by using individual ratings of anticipatory fear (fear during anticipation of severe dyspnea minus fear during anticipation of mild dyspnea) as covariate for the anticipation contrast (anticipation of severe dyspnea versus anticipation of mild dyspnea).

For statistical inference on our results, we used a two-step approach: first, we tested for significantly increased activation throughout the entire brain exceeding a whole-brain family-wise error corrected threshold of *p* < 0.05 within a cluster of more than 30 contiguous voxels. For the second analysis we chose the following bilateral regions of interest (ROIs): insula, anterior cingulate cortex, amygdala, and a midbrain-region including the PAG. Bilateral masks for insula, anterior cingulate cortex, and amygdala were generated from the automated anatomical labeling (AAL) template described by Tzourio-Mazoyer et al. [41]. AAL was also used to further specify the localization of cerebellar activation. A midbrain ROI centered on PAG was defined using an 8 mm sphere around the average coordinates for PAG activation reported by Linnman et al. [42]. The selection of these ROIs was based on results of previous studies on the anticipation of aversive stimuli including pain [26, 30, 31, 43–45]. Activation within each ROI was considered significant, if exceeding a threshold of *p* < 0.05 after family-wise error correction within the ROI.

## 3. Results

### 3.1. Respiratory Parameters and Dyspnea Ratings

Respiratory parameters showed significant variation across conditions (Table 2). As expected, post hoc *t*-tests showed significantly increased peak inspiratory mouth pressure and inspiratory time, as well as decreased breathing frequency during severe compared to mild dyspnea. During the two anticipation periods subjects showed similar breathing patterns with no significant differences in respiratory parameters except for *P*_ET_CO_2___, which was slightly lower during the anticipation of severe as compared to mild dyspnea.

**Figure 2:**
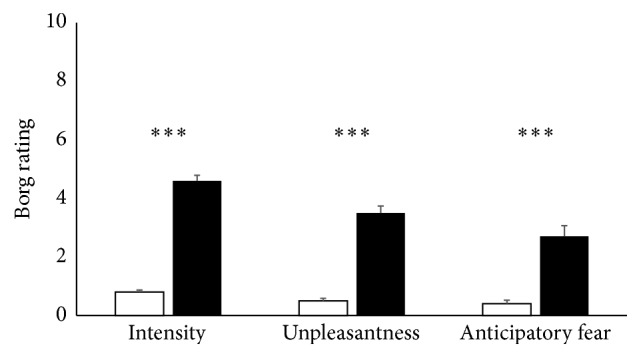
Mean (SEM) ratings of perceived intensity, unpleasantness, and anticipatory fear for mild (white) and severe (black) dyspnea, respectively. Increases from mild to severe dyspnea were significant at *p* < 0.001. *∗∗∗* indicates a significance level of *p* < 0.001.

Similarly, subjective dyspnea ratings confirmed successful induction of mild and severe dyspnea, respectively (Figure 2). Ratings for intensity and unpleasantness of resistive-load-induced dyspnea were significantly higher during severe (mean/SD = 4.6/1.3 and 3.5/1.6, resp.) than during mild dyspnea (mean/SD = 0.8/0.5 and 0.5/0.6, resp.). Likewise, the anticipatory fear was significantly stronger for severe as compared to mild dyspnea (mean/SD = 2.7/2.5 and 0.4/0.8, resp.). Verbal descriptor ratings revealed that resistive-load-induced dyspnea was mainly perceived as “increased work and effort of breathing.”

### 3.2. Functional Imaging Data

**Figure 3:**
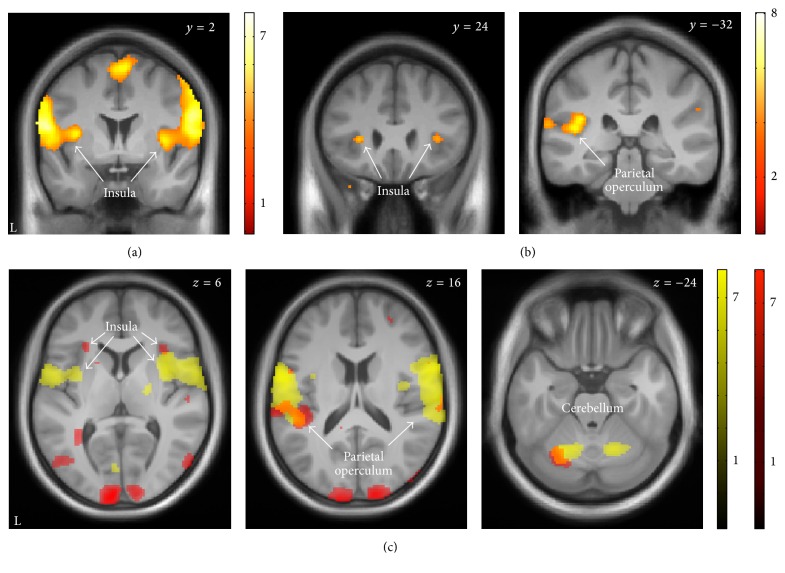
Perception (a) and anticipation (b) of severe versus mild dyspnea activated insular, parietal opercular, and cerebellar cortex. (c) Activation during anticipation (displayed in red) and perception of dyspnea (displayed in yellow) overlapped in the left cerebellum and parietal operculum while insular activation was more anterior during anticipation as compared to perception of dyspnea. Activation patterns are displayed at a threshold of *p* < 0.05, corrected for the specific ROI, and superimposed on the group-specific T1-weighted mean image generated by the DARTEL-protocol. L = left.

When contrasting the perception of severe dyspnea with mild dyspnea the whole-brain analysis confirmed the activation of a bilateral cortical network with activation peaks in pre- and postcentral cortices, SMA, parietal opercular cortex, cerebellum, and right insular cortex (Table 3). The ROI-based analysis yielded additional significant activation of the left insula (Figure 3(a), Table 3).

For anticipation of severe dyspnea versus anticipation of mild dyspnea the whole-brain analysis yielded significant activation that localized to the bilateral occipital pole and the left parietal operculum and cerebellum (Table 4). Further activation was found in bilateral insular cortex, which proved significant for the left anterior insular cortex in the ROI-based analysis (Figure 3(b), Table 4).

Activation during dyspnea anticipation and dyspnea perception showed substantial overlap within the parietal operculum and the cerebellum (6th cerebellar lobule), while insular activation during dyspnea anticipation was more anterior compared to insular activation during dyspnea perception (Figure 3(c)).

**Figure 4:**
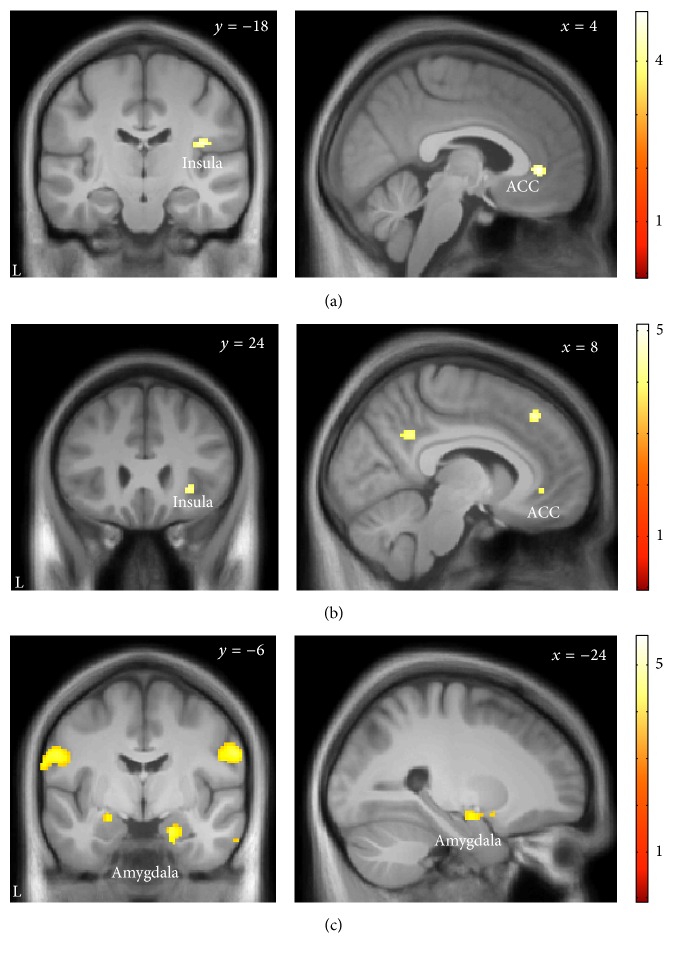
Psychophysiological interactions during the anticipation period. (a) During the anticipation of severe as compared to mild dyspnea the left anterior insular cortex shows significantly increased coactivation with ACC (*x* = 4, *y* = 38, *z* = 0; ROI-corrected *p* = 0.003) and right insular cortex (*x* = 36, *y* = −18, *z* = 18; ROI-corrected *p* = 0.019). (b) The left parietal operculum shows increased coactivation with ACC (*x* = 8, *y* = 40, *z* = −4; ROI-corrected *p* = 0.029) and right insular cortex (*x* = 30, *y* = 26, *z* = −4; ROI-corrected *p* = 0.007). (c) The left cerebellar cortex shows increased coactivation with the amygdala, bilaterally (*x* = −24, *y* = −8, *z* = −14; ROI-corrected *p* = 0.011 and *x* = 22, *y* = −4, *z* = −24; ROI-corrected *p* = 0.016, resp.). The nonsignificant interactions within the rostral cingulate cortex (b) and primary sensorimotor cortex (c) were outside our ROIs and did not reach whole-brain-corrected significance. All interactions are displayed at a threshold of *p* < 0.05, corrected for the specific ROI, and superimposed on the group-specific T1-weighted mean image generated by the DARTEL-protocol.

Next, we investigated the interactions between anterior insula, parietal operculum, and the 6th cerebellar lobule with other brain areas during the anticipation of dyspnea using PPIs. There were no significant interaction effects in the whole-brain analyses. However, the ROI-based approach showed significantly increased interactions of left anterior insula and parietal operculum during dyspnea anticipation with the right insular cortex and the ACC (Figures 4(a) and 4(b)). The left 6th cerebellar lobule showed a significantly increased interaction with bilateral amygdala (Figure 4(c)).

**Figure 5:**
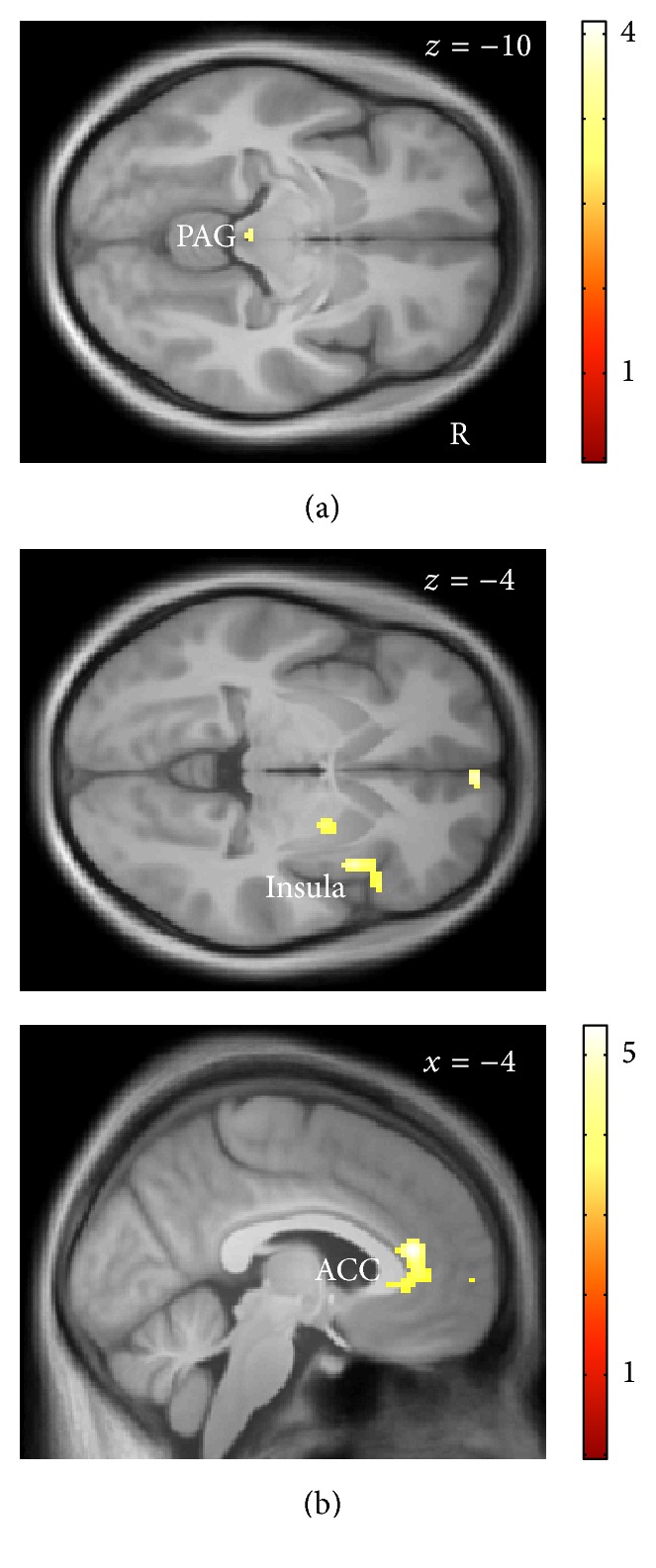
(a) Backward model: right insular activation during dyspnea perception is significantly correlated with midbrain/PAG activation during dyspnea anticipation (*x* = −2, *y* = −30, *z* = −10; ROI-corrected *p* = 0.026). (b) Anticipatory fear is significantly correlated with increased brain activation during dyspnea anticipation in the ACC (*x* = −4, *y* = 36, *z* = 14; ROI-corrected *p* = 0.001) and right insular cortex (*x* = 38, *y* = 12, *z* = −4; ROI-corrected *p* = 0.007). The nonsignificant correlations with the putamen and medial prefrontal cortex (b) were outside our ROIs and did not reach whole-brain-corrected significance. All correlations are displayed at a threshold of *p* < 0.05, corrected for the specific ROI, and superimposed on the group-specific T1-weighted mean image generated by the DARTEL-protocol.

Furthermore, we looked at the relationship of right insular activation during dyspnea perception with anticipatory brain activation. The ROI-based analysis showed that right insular activation during dyspnea perception was significantly correlated with activation in the midbrain/PAG during the anticipation of severe versus mild dyspnea (Figure 5(a)).

Ratings of anticipatory fear revealed a significant positive correlation with anticipatory brain activation within ACC and right insular cortex in the ROI-based analysis (Figure 5(b)).

## 4. Discussion

The present study investigated brain activations associated with the anticipation and perception of resistive-load-induced dyspnea in healthy subjects. Our analyses confirmed the involvement of a previously described set of brain areas for the perception of dyspnea [4, 16, 17]. This network included sensorimotor areas (pre- and postcentral gyri, SMA, and parietal operculum), bilateral insular cortex, and the cerebellum. Importantly, within the insular, parietal opercular, and cerebellar cortex activation was already increased during the anticipation of dyspnea. Anticipatory and dyspnea-related activation overlapped within parietal operculum and cerebellum, while activation within the insular cortex was more anterior during anticipation as compared to perception of resistive-load-induced dyspnea. During the anticipation of dyspnea, left anterior insula and parietal operculum showed increased connectivity with ACC and right insular cortex, while the cerebellum showed increased interaction with the bilateral amygdala. Notably, midbrain/PAG activation during dyspnea anticipation correlated with right insular activation during the subsequent perception of dyspnea. Finally, activation in the right insular cortex and ACC during the anticipation of dyspnea showed a significant positive correlation with anticipatory fear.

Taken together, the present study reveals prominent activation in several emotion-related brain areas during the anticipation of resistive-load-induced dyspnea, which were paralleled by increased anticipatory fear. Thus, both behavioral and functional brain data underline the relevance of affective processes during the anticipation of dyspnea, which in turn partly relate to the subsequent processing of perceived dyspnea.

The present findings converge with previous studies in several ways. First, conditioning studies demonstrated increased physiological fear responses during the anticipation of dyspneic breathing occlusions and hyperventilation including increased startle reflex magnitudes [21, 22]. The present study extends these findings to increased subjective fear reports and the involvement of fear-related brain areas during the anticipation of resistive-load-induced dyspnea.

Second, studies examining the anticipation of other aversive stimuli such as pain [23, 30], restricted breathing [40], negative affective pictures [43, 44], monetary loss [45], and hyperventilation cues [46] reported comparable activations in emotion-related brain areas as the present study. These included prominent activations in anterior insula, amygdala, anterior cingulate cortex, and PAG. These areas, especially amygdala and insula, have also been described as parts of a salience network for the detection of threatening stimuli [47].

Third, studies comparing anticipation with perception of aversive stimuli such as pain similarly demonstrated overlapping brain activation patterns [23–25, 30]. Furthermore, anticipatory brain activation had an influence on subsequent pain. More specifically, the prestimulus connectivity of anterior insula and midbrain/PAG [26] and anticipatory activation in anterior insula, anterior midcingulate cortex, and amygdala [48] have been found to influence both, brain activation during actual pain perception and behavioral markers of pain.

Fourth, previous studies have linked activation in insula and extended amygdala to the affective unpleasantness of dyspnea [11, 40]. This supports the suggested relevance of these two brain areas for affective responses (e.g., fear) towards upcoming dyspnea, which is further supported by the present correlation between anticipatory fear ratings and anticipatory insular activity.

Finally, overlapping activation for dyspnea anticipation and perception localized to the 6th cerebellar lobule. This cerebellar subdivision has been shown in neuroimaging and lesion studies to be relevant for emotional processes [49, 50] including the processing of different aversive stimuli such as pain and negative affective pictures [51]. Although cerebellar activation has frequently been reported for various dyspneic stimuli [8, 9, 52–54], its particular contribution to dyspnea perception is only poorly understood. The present observation of anticipatory activity paralleled by strong amygdala interactions is in line with a previously suggested involvement in both, sensorimotor and affective aspects of dyspnea [17].

The present findings suggest a neural correlate for the recently proposed link between anticipatory fear and later avoidance behavior as one underlying cause of negative health outcome in chronic dyspnea [20]. Several clinical studies [6, 55] have demonstrated that dyspnea specific fears or worries about physical exercise are related to worse performance in exercise tests and worse outcome of pulmonary rehabilitation in patients with COPD. These findings suggest that irrespective of disease severity anticipatory fear of dyspnea leads to unfavorable health behavior such as avoidance of higher exercise levels. A similar connection between fear of bodily symptoms (e.g., lower back pain), avoidance behavior, and subsequent negative course of disease has already been established in the fear avoidance model of pain [56]. Notably, pain research has already demonstrated the beneficial effects of cognitive behavioral treatments for reducing anticipatory fear of pain and improving the course of disease [57].

Although dyspnea and pain are processed by distinct neural pathways, similarities concerning the emotion-related processes during the anticipation of aversive bodily sensation can be assumed [12]. Therefore, adapting these treatments that have been proven successful in chronic pain to the treatment of anticipatory fear of dyspnea in patients suffering from chronic dyspnea seems promising [58]. Findings of reduced gray matter volume in, for example, ACC and amygdala, in patients suffering from chronic obstructive pulmonary disease [59] provide first evidence that chronic dyspnea indeed impacts the neural structure of emotion-related areas, potentially related to anticipatory fear.

The following limitations of the present study should be kept in mind: To keep the contingency of anticipation and dyspnea periods, anticipatory fear was not assessed immediately after each anticipation cue. A prompt rating after the anticipation period would certainly allow a more precise assessment of anticipatory fear and would also reflect potential fluctuations over time. Next, we investigated resistive-load-induced dyspnea causing a sensation of increased work and effort of breathing. Thus, our results cannot be generalized to other qualities of dyspnea such as air hunger and chest tightness. Furthermore, our data on predominantly young healthy subjects cannot be generalized to older subjects in general and subjects suffering from chronic dyspnea in particular. Therefore, further research is needed to investigate whether anticipatory fear and respective brain activation patterns within our experimental setting are suitable approximations to understand avoidance behavior in everyday life including reduced physical activity in patients suffering from chronic dyspnea. Respective findings would open a new avenue to behavioral training aimed at reducing anticipatory fear of dyspnea in the treatment of chronic dyspnea.

## 5. Conclusions

Dyspnea anticipation and perception share a similar set of brain areas. Furthermore, anticipatory midbrain/PAG activation was associated with subsequent dyspnea-related activation of the insular cortex. During dyspnea anticipation the prominent involvement of emotion-related areas such as insula, ACC, and amygdala is suggested as potential correlate of anticipatory fear of dyspnea, which might underlie the development of unfavorable health behaviors in patients suffering from dyspnea.

## Figures and Tables

**Table 1:** Mean (SD) baseline characteristics of subjects.

Age (yr)	28.5 (6)
Sex (female/male), *n*	18/28
Weight (kg)	75.5 (12.9)
Height (cm)	178.9 (9.6)
Body mass index (kg/m^2^)	23.4 (2.4)
FEV_1_ (L)	4.77 (0.98)
FEV_1_ (% predicted)	115.3 (12.6)
FVC (L)	5.73 (1.27)
FVC (% predicted)	117.04 (13.14)

FEV_1_ = forced expiratory volume in 1 s, FVC = forced vital capacity.

**Table 2:** Group mean (SD) respiratory parameters for conditions of anticipation and perception of dyspnea.

	Anticipation of dyspnea		Perception of dyspnea	
	Mild	Severe	Mild	Severe
*P*_ET_CO_2___ (mmHg)	33.81 (3.46)	33.45 (3.4)^*∗*^	33.82 (3.4)	33.85 (3.6)
*f* (breaths/min)	13.81 (3.66)	13.75 (3.4)	13.69 (3.63)	12.72 (3.7)^†^
*P*_*I*_ (mbar)	1.11 (0.31)	1.05 (0.3)	2.27 (0.89)	9 (3.97)^†^
*T*_*I*_ (s)	1.83 (0.61)	1.79 (0.41)	2.06 (0.56)	2.43 (0.67)^†^
*V*_*E*_ (L/min)	10.04 (2.34)	9.56 (2.56)	12.03 (2.93)	11.48 (2.94)
*V*_*T*_ (L)	0.82 (0.35)	0.77 (0.3)	0.95 (0.31)	1 (0.39)

^*∗*^ *p* corrected < 0.05, ^†^ *p* corrected < 0.01 for anticipation of severe versus anticipation of mild and severe dyspnea versus mild dyspnea, respectively.

*P*_ET_CO_2___ = end-tidal CO_2_ pressure; *f* = breathing frequency; *P*_*I*_ = peak inspiratory mouth pressure; *T*_*I*_ = inspiratory time; *V*_*E*_ = minute ventilation; *V*_*T*_ = tidal volume.

**Table 3:** Peak coordinates, *t*-statistics, and uncorrected *p* values for significant brain activations during the perception of severe versus mild dyspnea.

Area	*x*	*y*	*z*	*t*	*p*
Precentral cortex R	44	−8	38	7.02	<0.001^*∗*^
	56	4	14	6.2	<0.001^*∗*^
Precentral cortex L	−42	−12	40	7.73	<0.001^*∗*^
	−60	−28	18	6.7	<0.001^*∗*^
Postcentral cortex R	46	−10	36	7.3	<0.001^*∗*^
	66	−18	20	6.3	<0.001^*∗*^
Postcentral L	−42	−12	34	7.11	<0.001^*∗*^
Supplementary motor area R	4	−24	62	5.75	<0.001^*∗*^
Supplementary motor area L	−4	−2	60	5.46	<0.001^*∗*^
Parietal operculum (SII) R	64	−18	20	6.31	<0.001^*∗*^
Parietal operculum (SII) L	−54	−16	20	6.41	<0.001^*∗*^
Insula R	36	6	4	5.78	<0.001^*∗*^
Insula L	−36	0	10	5.05	<0.001^†^
Cerebellar hemisphere R	14	−62	−20	5.41	<0.001^*∗*^
Cerebellar hemisphere L	−26	−62	−24	6.72	<0.001^*∗*^

^*∗*^Whole-brain family-wise error corrected *p* < 0.05.

^†^Small volume corrected *p* for respective bilateral ROI < 0.05.

*x* = left-right coordinate, *y* = posterior-anterior coordinate, *z* = inferior-superior coordinate, R = right hemisphere; L = left hemisphere.

**Table 4:** Peak coordinates (*x*, *y*, *z*), *t*-statistics, and uncorrected *p* values for significant brain activations during the anticipation of severe versus mild dyspnea.

Area	*x*	*y*	*z*	*t*	*p*
Occipital pole R	14	−96	12	7.14	<0.001^*∗*^
Occipital pole L	−8	−100	8	8.00	<0.001^*∗*^
Parietal operculum L	−40	−32	20	5.54	<0.001^*∗*^
Insula L	−30	26	4	4.18	<0.001^†^
Cerebellar hemisphere L	−28	−64	−24	5.79	<0.001^*∗*^

^*∗*^Whole-brain family-wise error corrected *p* < 0.05.

^†^Small volume corrected *p* for respective bilateral ROI < 0.05.

*x* = left-right coordinate, *y* = posterior-anterior coordinate, *z* = inferior-superior coordinate, R = right hemisphere, and L = left hemisphere.
